# Synthetic 4-Hydroxy Auxarconjugatin B, a Novel Autophagy Inducer, Attenuates Gouty Inflammation by Inhibiting the NLRP3 Inflammasome

**DOI:** 10.3390/cells9020279

**Published:** 2020-01-23

**Authors:** Chih-Yu Hsieh, Lan-Hui Li, Yulin Lam, Zhanxiong Fang, Chin Heng Gan, Yerra Koteswara Rao, Hsiao-Wen Chiu, Wei-Ting Wong, Tz-Chuen Ju, Fang-Hsin Chen, Oleg V. Chernikov, May-Lan Liu, Chung-Hua Hsu, Kuo-Feng Hua

**Affiliations:** 1 Department of Biotechnology and Animal Science, National Ilan University, Ilan 260, Taiwan; fish37435@hotmail.com (C.-Y.H.); raoyk81@gmail.com (Y.K.R.); looking123123@hotmail.com (H.-W.C.); non8908@gmail.com (W.-T.W.); 2 Department of Laboratory Medicine, Linsen, Chinese Medicine and Kunming Branch, Taipei City Hospital, Taipei 10844, Taiwan; A1525@tpech.gov.tw; 3 Department of Pathology, Tri-Service General Hospital, National Defense Medical Center, Taipei 11483, Taiwan; 4 Department of Chemistry, National University of Singapore, 3 Science Drive 3, Singapore 117543, Singapore; chmlamyl@nus.edu.sg (Y.L.); fangzhanxiong@gmail.com (Z.F.); a0072125@u.nus.edu (C.H.G.); 5 Department of Animal Science and Biotechnology, Tunghai University, Taichung 407, Taiwan; tzchuen@thu.edu.tw; 6 Department of Medical Imaging and Radiological Sciences, Chang Gung University, Taoyuan 33302, Taiwan; fanghsinchen@mail.cgu.edu.tw; 7 G.B. Elyakov Pacific Institute of Bioorganic Chemistry FEB RAS, Vladivostok 690022, Russia; chernikovoleg@gmail.com; 8 Department of Nutritional Science, Toko University, Chiayi 61363, Taiwan; 2009nh02@mail.toko.edu.tw; 9 Institute of Traditional Medicine, School of Medicine, National Yang-Ming University, Taipei 11221, Taiwan; owlherbs@yahoo.com.tw; 10 Department of Chinese Medicine, Linsen, Chinese Medicine and Kunming Branch, Taipei City Hospital, Taipei 10844, Taiwan; 11 Department of Medical Research, China Medical University Hospital, China Medical University, Taichung 40402, Taiwan

**Keywords:** 4-hydroxy auxarconjugatin B, NLRP3 inflammasome, mitochondria, autophagy, gouty inflammation

## Abstract

Gouty arthritis results from the generation of uric acid crystals within the joints. These uric acid crystals activate the NACHT, LRR and PYD domains-containing protein 3 (NLRP3) inflammasome, which is involved in chronic inflammatory diseases, including gouty arthritis. This study identified the polyenylpyrrole derivative 4-hydroxy auxarconjugatin B (4-HAB), a novel autophagy inducer, which attenuated uric acid crystals-mediated activation of the NLRP3 inflammasome in vitro and in vivo. 4-HAB dose-dependently reduced the release of interleukin (IL)-1β, IL-18, active caspase-1 and apoptosis-associated speck-like protein (ASC) in uric acid crystals-activated macrophages. In a mechanistic study, 4-HAB was shown to inhibit uric acid crystals-induced mitochondrial damage, lysosomal rupture and ASC oligomerization. Additionally, 4-HAB inhibited the NLRP3 inflammasome through Sirt1-dependent autophagy induction. Furthermore, the anti-inflammatory properties of 4-HAB were confirmed in a mouse model of uric acid crystals-mediated peritonitis by the reduced levels of neutrophil influx, IL-1β, active caspase-1, IL-6 and MCP-1 in lavage fluids. In conclusion, 4-HAB attenuates gouty inflammation, in part by attenuating activation of the NLRP3 inflammasome through the Sirt1/autophagy induction pathway.

## 1. Introduction

Macrophages are one of the important immune cells that play an essential role in the host defence system; however, excessive inflammation caused by over reactive macrophages and leads to inflammatory diseases or autoimmune disorders [1]. Unlike traditional proinflammatory cytokines, interleukin (IL)-1β and IL-18 production are regulated by post-translational modification catalysed by protease caspase-1. The caspase-1 activity is regulated by a group of cytoplasmic multiprotein complexes called inflammasomes [2]. The NACHT, LRR and PYD domains-containing protein 3 (NLRP3) inflammasome is the best-characterized inflammasome composed of NLRP3, apoptosis-associated speck-like protein (ASC) and caspase-1. The NLRP3 inflammasome recognizes and responses to diverse pathogen associated molecular patterns from infectious microorganisms or sterile danger signals generated by host cells [2]. IL-1β and IL-18 produced by inflammasomes not only activate innate immunity but also influence T cell adaptive immunity [3].

The NLRP3 inflammasome activation is dependent on the priming and activation signals. The priming signal is triggered mainly by the Toll-like receptors and induces the gene and protein expressions of NLRP3 and IL-1β precursor (proIL-1β) through reactive oxygen species (ROS)-, mitogen-activated protein kinase (MAPK)- and nuclear factor kappa B (NF-κB)-dependent pathways [4]. Only priming signal, however, is insufficient to activate the NLRP3 inflammasome unless the cells are activated by the activation signal, such as gout-causing monosodium urate (MSU) crystals, diabetes-causing saturated fatty acids, Alzheimer’s disease-causing amyloid-β and bacterial toxin nigericin [5]. The activation signals trigger the downstream signalling events, such as lysosomal destabilization and mitochondrial dysfunction that induce NLRP3 inflammasome assembly and eventually caspase-1 activation [2]. As the NLRP3 inflammasome recognizes and responses to the board range medicinally relevant stimuli, dysregulated NLRP3 inflammasome activation participates many human inflammatory diseases, including gout, type II diabetes, atherosclerosis and neurodegenerative disorders [5]. Therefore, targeting the NLRP3 inflammasome has therapeutic significance in the management of dysregulated NLRP3 inflammasome complications.

**Figure 1:**
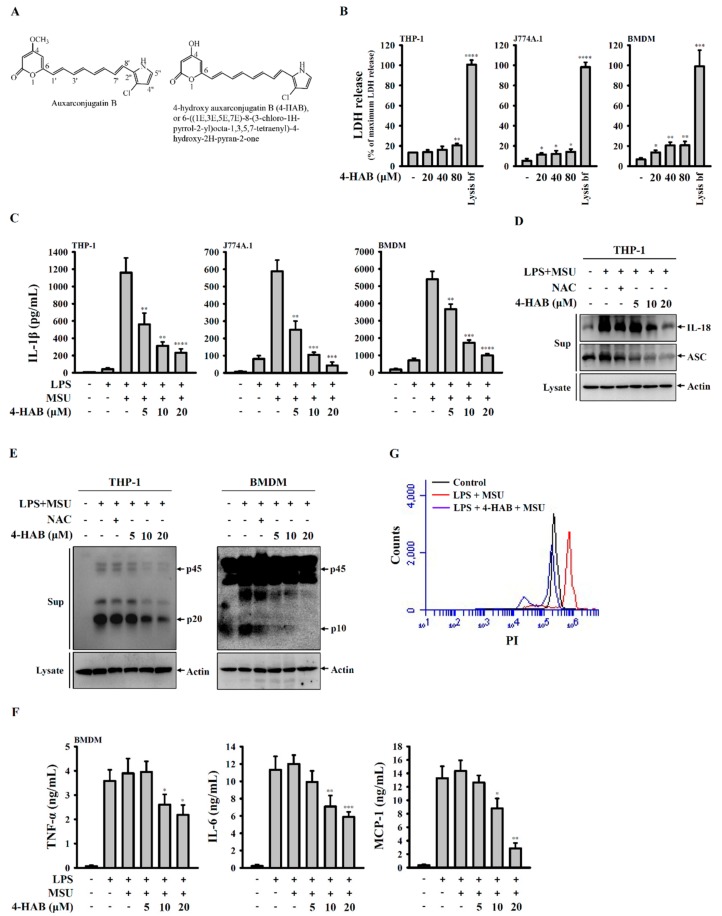
4-HAB reduced the NACHT, LRR and PYD domains-containing protein 3 (NLRP3) inflammasome activation in MSU crystal-activated macrophages. (**A**) Chemical structure of 4-HAB. (**B**) Cells were incubated with 4-HAB for 24 h, and cytotoxicity was analyzed by LDH release. (**C**–**G**) Cells were incubated with 1 µg/mL LPS for 5 h followed by incubated with 4-HAB for 30 min. Cells then incubated with 100 μg/mL MSU crystals for additional 24 h. The control group was treated with vehicle control. The levels of IL-1β in the supernatants were measured by ELISA (**C**); the levels of IL-18 and ASC in the supernatants were measured by Western blot (**D**); the levels of active caspase-1 (p20 or p10) in the supernatants were measured by Western blot (**E**); the levels of TNF-α, IL-6 and MCP-1 in the supernatants were measured by ELISA (**F**); the PI uptake by THP-1 macrophages was measured by flow cytometry (**G**). The data are expressed as the mean ± SD of three separate experiments. *, **, *** and **** indicate a significant difference at the level of *p* < 0.05, *p* < 0.01, *p* < 0.001 and *p* < 0.0001, respectively, compared to control (**B**) or MSU crystals/LPS-treated cells. (One-way ANOVA with Dunnett’s multiple comparisons test). “+” indicates with; “−“ indicates without.

The current therapeutic strategies for NLRP3-associated complications are based on the non-steroidal anti-inflammatory drugs, colchicines or glucocorticoids, which are not satisfactory and cause significant side effects [6]. Therefore, the development of a novel NLRP3 inflammasome inhibitor is a therapeutic option to counteract dysregulated NLRP3-associated diseases. Conjugated polyenes are an interesting class of widely occurring natural products. Previous reports on conjugated polyenes indicate that they have various biological properties, such as their antibacterial, antifungal, and antitumour activities [7]. Presently, some conjugated polyenes, including rapamycin and fumagillin, are commercially available. Clark et al. previously reported the isolation and structure elucidation of several polyenylfurans and polyenylpyrroles from the soil microbe *Gymnoascus reessii* [8]. We have also shown that the related polyenes auxarconjugatins A and B, which contain a chloropyrrole group, possess cytotoxic properties [9], whereas furan-containing gymnoconjugatins possess no significant activity [8]. The auxarconjugatin B derivative 4-hydroxy auxarconjugatin B, or 6-((1E,3E,5E,7E)-8-(3-chloro-1H-pyrrol-2-yl)octa-1,3,5,7-tetraenyl)-4-hydroxy-2H-pyran-2-one (4-HAB, Figure 1A), is a novel, low-molecular-weight polyenylpyrrole agent [9]. Our previous data showed that 4-HAB exerts strong anti-inflammatory effects by inhibiting lipopolysaccharide (LPS)-induced inflammation in macrophages and dendritic cells [10]. However, little is known about the effects of 4-HAB on the NLRP3 inflammasome and the underlying molecular mechanism of these effects. As part of our efforts is to identify novel NLRP3 inflammasome inhibitors [11,12,13,14,15] and based on the known anti-inflammatory effects of 4-HAB, we hypothesized that 4-HAB can inhibit the NLRP3 inflammasome.

## 2. Materials and Methods

### 2.1. Reagents and Chemicals

*Escherichia coli* 0111:B4 lipopolysaccharide (LPS), N-acetyl-L-cysteine (NAC), acridin orange (AO), monodansylcadaverine (MDC), 3-Methyladenine (3-MA), 6-Chloro-2,3,4,9-tetrahydro-1H-Carbazole-1-carboxamide (EX-527), phorbol myristate acetate (PMA) and propidium iodide (PI) and uric acid were purchased from Sigma-Aldrich (St. Louis, MO, USA). Rapamycin and puromycin were purchased from InvivoGen (San Diego, CA, USA). GeneJammer^®^ transfection reagent was purchased from Agilent Technologies (Santa Clara, CA, USA). Antibodies against human IL-1β, ASC, IL-18, Actin and horseradish peroxidase-labeled secondary antibodies were obtained from Santa Cruz Biotechnology (Santa Cruz, CA, USA). Antibodies against human caspase-1 were obtained from Cell Signaling Technology (Beverly, MA, USA). Antibodies against NLRP3 and mouse caspase-1 were purchased from Adipogen International (San Diego, CA, USA). Antibody against mouse IL-1β was purchased from R&D systems (Minneapolis, MN, USA). Antibody against LC3B was purchased from Novus Biologicals (Littleton, CO, USA). Antibodies against Gr1 and CD45 were purchased from eBioscience (San Diego, CA, USA). JC-1 and Antibodies against Cathepsin B and Sirt1 were purchased from Millipore (Bedford, MA, USA). MitoTracker Deep Red, MitoTracker Green, MitoSOX and Pierce™ LAL Chromogenic Endotoxin Quantitation Kit were purchased from Thermo Scientific (Rockford, IL, USA). Magic Red Cathepsin B detection kit was purchased from ImmunoChemistry Technologies (Bloomington, MN, USA). The CytoScan LDH Cytotoxicity Assay kit was purchased from G-Bioscience (St. Louis, MO, USA).

### 2.2. Cell Lines and Culture

The murine J774A.1 macrophages and human THP-1 monocytes were purchased from the American Type Culture Collection (Rockville, MD, USA) and cultured in RPMI 1640 medium contained with 10% heat-inactivated fetal bovine serum at 37 °C in a 5% CO_2_ incubator. To induce monocytes differentiation into macrophages, THP-1 monocytes were treated with 50 nM PMA for 48 h. Non-adherent cells were removed by aspiration, and adherent macrophages were washed with RPMI 1640 medium before stabilizing for additional 48 h in cell culture medium. Bone marrow-derived macrophages (BMDM) were prepared from marrow collected from C57BL/6 mice femur and tibia incubated for seven days in culture medium containing M-CSF (Peprotech, London, UK). For generating gene knockout cells, cells were transfected with CRISPR/Cas9 knockout plasmids targeting LC3 (for J774A.1 macrophages: sc-426563 and sc-417828-HDR, Santa Cruz Biotechnology; for THP-1 monocytes: sc-4178288 and sc-417828-HDR, Santa Cruz Biotechnology) or Sirt1 (for J774A.1 macrophages: sc-430046 and sc-430046-HDR, Santa Cruz Biotechnology). The CRISPR/Cas9 knockout plasmids transfected cells were selected by puromycin and the expression levels of LC3 and Sirt1 were checked by Western blot.

### 2.3. General Procedure for the Synthesis of 4-hydroxy Auxarconjugatin B, or 6-((1’E,3’E,5’E,7’E)-8’-(3-chloro-1H-pyrrol-2-yl)octa-1,3,5,7-tetraenyl)-4-hydroxy-2H-pyran-2-one (4-HAB)

Compound 4-HAB was synthesized according to the experimental procedures which we have reported previously [9]. Briefly, tris(dibenzylideneacetone)dipalladium(0) (Pd2(dba)3) (2.7 mg, 3.0 μmol) and triphenylarsine (AsPh_3_) (4.6 mg, 15 μmol) were added to dry THF (1 mL) followed by 2-(2-bromovinyl)-3-chloro-1-(methylsulfonyl)-1H-pyrrole (56 mg, 0.195 mmol) and 1.8 M aqueous KOH (0.167 mL, 0.300 mL). 4-Hydroxy-6-((1E,3E,5E)-6-(4,4,5,5-tetramethyl-1,3,2-dioxaborolan-2-yl)hexa-1,3,5-trienyl)-2H-pyran-2-one (0.15 mmol) dissolved in THF (0.5 mL) was then added dropwise to the reaction mixture over 5 min with stirring. The reaction mixture was stirred for 20 min at room temperature and quenched with saturated NH_4_Cl. The reaction mixture was extracted with ethyl acetate (EtOAc), the organic layer dried over MgSO_4_ and concentrated under reduced pressure. The residue obtained was dissolved in THF (1 mL), and 1 M tetra-n-butylammonium fluoride (TBAF) in THF (0.300 mL, 0.300 mmol) was added. The mixture was stirred at room temperature for 30 min, and thereafter, EtOAc was added and the mixture was washed thrice with water followed by saturated NaCl solution. The organic layer was dried over MgSO_4_, concentrated under reduced pressure, and purified by column chromatography. Compound 4-HAB was characterized by 1H and 13C NMR and mass spectral data, which were identical to the previously reported values [9].

### 2.4. MSU Crystals Preparation

MSU crystals were prepared according to the method described previously [16]. In brief, 250 mg uric acid was dissolved in 45 mL of boiling water containing 0.3 mL of 5 M NaOH. After the solution was passed through a 0.2-µM filter, 1 mL of 5 M NaCl was added to the solution, and keeps the solution at 26 °C for 7 days. The resulting crystals collected and washed with ethanol and acetone. The crystals evaporated and sterilized by heating at 180 °C for 2 h and stored in a sterile environment until use. All MSU crystals were determined to be endotoxin free (<0.01 EU/10 mg) by the LAL assay.

### 2.5. Cytokines and Proteins Measurements

The levels of cytokines in the culture medium and peritoneal lavage fluids were measured by Enzyme-Linked ImmunoSorbent Assay (ELISA) as described in our previous study [10]. For detection of protein expression in the culture medium, the medium was concentrated by the following protocol before performing Western blot [11]. Briefly, prepared a mixture containing 400 µL culture medium, 400 µL methanol and 166 µL chloroform. The mixture was vortexed and 400 µL double-distilled water was added. The mixture was thoroughly vortexed and incubated on ice for 10 min before centrifugation at 13,000 rpm at 4 °C for 10 min. The supernatant was removed and mixed the pellet with 500 µL methanol before centrifugation at 13,000 rpm at 4 °C for 10 min. The supernatant was removed and the pellet was dried at 55 °C and dissolved in Western blot loading buffer, followed by incubation in boiling water for 30 min. The samples were analysed by Western blot as described in our previous study [10]. For detection of protein expression in the lysates, the cells were washed with ice-cold phosphate-buffered saline (PBS) and lysed with 100 μL ice-cold lysis buffer (20 mM Tris-HCl (pH 7.5), 150 mM NaCl, 1 mM EDTA, 1 mM EGTA, 1% NP-40, 1% sodium deoxycholate, 2.5 mM sodium pyrophosphate, 1 mM β-glycerolphosphate, 1 mM Na_3_VO_4_, 1 μg/mL leupeptin and 1 mM PMSF) on ice for 10 min. The samples were pelleted by centrifuging at 12,000× *g* at 4 °C for 15 min, and the protein concentrations of supernatants were determined using Bio-Rad protein assay dye. 50 μg proteins form each sample was analysed by Western blot as described in our previous study [10].

### 2.6. ASC Oligomerization and Speck Formation

ASC oligomerization was analyzed by Western blot-based assay. In brief, insoluble ASC complexes were isolated from cell lysates by centrifugation and subsequent crosslinking with disuccinimidylsuberate as previously described [17]. The samples were resolved on 15% sodium dodecyl sulfate polyacrylamide gel electrophoresis and processed for Western blot. For ASC speck formation, the cells were fixed in pre-warmed 4% paraformaldehyde (PFA) in PBS for 30 min, and then permeabilized by 0.2% Triton X-100. After blocking with 1% bovine serum albumin, ASC speck formation was measured by incubating the cells with ASC antibody and fluorescent-conjugated secondary antibody. The cells were visualized by an Olympus BX-41 microscope and the images were analyzed by Image J.

### 2.7. Autophagy Measurement by AO and MDC Staining

THP-1 macrophages were incubated for 24 h with 20 µM 4-HAB or 100 nM rapamycin. The cells were stained with 1 µg/mL AO or 50 µM MDC at 37 °C for 10 min followed by washing with PBS twice, and then fixed in pre-warmed 4% PFA in PBS for 30 min. In the AO stained cells, the nucleus was stained by 4′,6-diamidino-2-phenylindole (DAPI). The cells were visualized by Olympus FV 1000-IX81 confocal microscope.

### 2.8. Cathepsin B Activity Assay

Cathepsin B activity were measured according to the product instruction manual of Magic Red Cathepsin B detection kit. In brief, THP-1 macrophages were incubated for 5 h with or without 1 μg/mL LPS followed by incubated with or without 20 µM 4-HAB for 30 min, then for 24 h with or without 100 μg/mL MSU crystals. The cells were stained for 10 min with Magic Red Cathepsin B detection kit at 37 °C, and then fixed for 30 min in pre-warmed 4% PFA in PBS. The cells were visualized by an Olympus FV 1000-IX81 confocal microscope.

### 2.9. Mitochondrial Function

THP-1 macrophages were incubated with or without 1 μg/mL LPS for 5 h, followed by incubated with or without 20 µM 4-HAB for 30 min, then with or without 100 μg/mL MSU crystals for 24 h. To measure the inner transmembrane potential and mitochondrial mass, cells were stained with 25 nM MitoTracker Deep Red and 25 nM MitoTracker Green for 15 min. To measure the mitochondrial ROS, cells were stained with 5 μM MitoSOX for 15 min. The degree of mitochondrial membrane polarization in THP-1 macrophages was measured by staining with 2 μM JC-1. The signals of MitoTracker Deep Red, MitoTracker Green and MitoSOX were detected by flow cytometry, and signals of JC-1 were detected by an Olympus FV 1000-IX81 confocal microscope.

### 2.10. Cell Membrane Integrity Assay

THP-1 macrophages were incubated for 5 h with or without 1 μg/mL LPS followed by incubated with or without 20 µM 4-HAB for 30 min, then for 24 h with or without 100 μg/mL MSU crystals. To measure the cell membrane integrity, cells were fixed by 70% ethanol and stained with 2 μg/mL PI for 15 min. The signals of PI were detected by flow cytometry.

### 2.11. LDH Release Assay

Cells were incubated with 4-HAB, 10% H_2_O (spontaneous LDH release) or lysis buffer (maximum LDH release) for 24 h. To determine LDH release, culture medium was evaluated for the presence of the LDH using the CytoScan LDH Cytotoxicity Assay kit according to the manufacturer’s instructions. Briefly, prepared a mixture containing 50 µL culture medium and 50 µL LDH substrate. The mixture was incubated in dark for 30 min before 50 µL stop buffer was added. The LDH release was assessed by measuring the optical density (OD) at 490/680 nm using a microplate absorbance reader. The cytotoxicity % was calculated as 100× (sample OD—spontaneous OD)/(maximum OD—spontaneous OD).

### 2.12. In Vivo Mice Model of MSU Crystals-Induced Peritonitis

Male C57BL/6JNal mice aged eight weeks old were purchased from The National Laboratory Animal Center (Taipei, Taiwan). The mice housed in a room controlled for temperature (23 ± 3 °C) and relative humidity (40%–60%). Mice were acclimated in the animal facility for at least a week before the experiments. Animal experiments were performed with the approval of the Institutional Animal Care and Use Committee of the National Ilan University (approval number: No. 102-40), according to the NIH Guide for the Care and Use of Laboratory Animals. The mice were randomized into four groups: Group I: control, i.p. injection of 0.5% DMSO in sterile PBS (200 μL) at 0, 24 and 48 h; i.p. injection of sterile PBS (0.5 mL) at 1 and 49 h, *n* = 6. Group II: MSU crystals treatment, i.p. injection of 0.5% DMSO in sterile PBS (200 μL) at 0, 24 and 48 h; i.p. injection of sterile MSU crystals (3 mg in 0.5 mL PBS) at 1 and 49 h, *n* = 8. Group III: 4-HAB+MSU crystals treatment, i.p. injection of 4-HAB (20 mg/kg body weight) at 0, 24 and 48 h; i.p. injection of sterile MSU crystals (3 mg in 0.5 mL PBS) at 1 and 49 h, *n* = 6. Group IV: Colchicine+MSU crystals treatment, i.p. injection of colchicine (1 mg/kg body weight) at 48 h; i.p. injection of sterile MSU crystals (3 mg in 0.5 mL PBS) at 1 and 49 h, *n* = 6. Mice were euthanized at 53 h and the peritonea were lavaged with 3 mL ice-cold PBS. The absolute number of cells obtained counted in a hemocytometer before staining them with Gr1 and CD45 antibodies and analyzed by flow cytometry. All analysis was performed using BD CSampler Software (version 227). The expression levels of cytokines in peritoneal lavage fluids were measured by ELISA.

### 2.13. Statistical Analysis

GraphPad Prism 7.0 software was used for data analysis. Data are shown as mean ± SEM. Statistical significance was determined by *t* tests (two-tailed) for two groups or ANOVA (with Dunnett’s multiple comparisons test) for three or more groups. *p* values less than 0.05 were considered to be statistically significant.

## 3. Results

### 3.1. 4-HAB Reduced MSU Crystals-Mediated Activation of the NLRP3 Inflammasome in LPS-Primed Macrophages

In our previous study we demonstrated that 4-HAB did not have significant cytotoxicity in mouse RAW 264.7 macrophages (IC50 > 100 µM) [9]. In this study we further investigated the cytotoxicity of 4-HAB in THP-1 macrophages, J774A.1 macrophages and BMDM by LDH release assay (Figure 1B). In THP-1 macrophages, the basal LDH release level in control cells was 13.3% ± 0.1% of maximum LDH release. 4-HAB at 20 and 40 µM (24 h treatment) did not increase LDH release, but slightly increased LDH release at 80 µM (20.5% ± 1.8%). In J774A.1 macrophages, the basal LDH release level in control cells was 5.1% ± 2.1% of maximum LDH release. 4-HAB at 20, 40 and 80 µM (24 h treatment) slightly increased LDH release to 11.4% ± 1.5%, 12.1% ± 3.1% and 14.1% ± 2.7%. In BMDM, the basal LDH release level in control cells was 6.6% ± 1.6% of maximum LDH release. 4-HAB at 20, 40 and 80 µM (24 h treatment) slightly increased LDH release to 13.5% ± 2.2%, 20.5% ± 3.2% and 20.6% ± 3.8%. These results indicated that 4-HAB did not have significant cytotoxicity in macrophages at concentration less than 80 µM. The maximum dose of 4-HAB we used in the following studies was 20 µM. We examined the effect of 4-HAB on MSU crystals-mediated activation of the NLRP3 inflammasome in LPS-primed macrophages. As can be seen in Figure 1C, LPS treatment (priming) slightly increased IL-1β secretion from 8.9 ± 1.6 to 41.6 ± 14.8 pg/mL (THP-1 macrophages), from 7.2 ± 4.8 to 80.9 ± 19.5 pg/mL (J774A.1 macrophages) and from 181.6 ± 46.9 to 705.8 ± 127.8 pg/mL (BMDM). MSU crystals treatment further significantly increased IL-1β secretion to 1160.2 ± 170.5 pg/mL (THP-1 macrophages), 588.1 ± 64.9 pg/mL (J774A.1 macrophages) and 5402.5 ± 456.9 pg/mL (BMDM). 4-HAB treatment dose-dependently reduced MSU crystals-induced IL-1β secretion in THP-1 macrophages, J774A.1 macrophages and BMDM (Figure 1C). Additionally, Western blotting indicated that 4-HAB dose-dependently inhibited the release of IL-18 and ASC in MSU crystals-activated THP-1 macrophages (Figure 1D). Next, we found that 4-HAB dose-dependently attenuated the release of active caspase-1 in MSU crystals-activated THP-1 macrophages and BMDM, as determined by Western blot (Figure 1E). Furthermore, 4-HAB also dose-dependently reduced the release of TNF-α, IL-6 and MCP-1 in LPS and MSU crystals-activated BMDM (Figure 1F). Pyroptosis is a type of caspase-1-dependent cell death characterized by the loss of cell membrane integrity [18]. We determined the uptake of PI to examine membrane damage in THP-1 macrophages during MSU crystals exposure. MSU crystals induced an increase in PI uptake in LPS-primed THP-1 macrophages, indicating the induction of pyroptosis. However, this effect was abolished by 4-HAB (Figure 1G). Taken together, our results indicated that 4-HAB is a putative inhibitor of the NLRP3 inflammasome in MSU crystals-activated macrophages.

### 3.2. 4-HAB Prevented Mitochondrial Damage and Increased Mitochondrial Biogenesis

**Figure 2:**
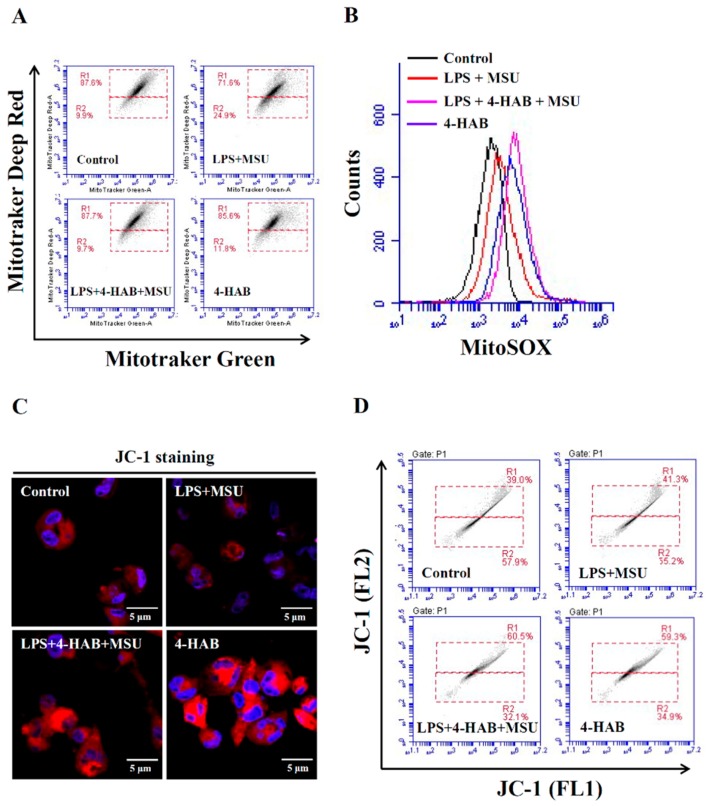
4-HAB prevented mitochondrial damage and increased mitochondrial biogenesis. THP-1 macrophages were incubated with 1 µg/mL LPS for 5 h followed by incubated with 20 µM 4-HAB for 30 min. Cells then incubated with 100 μg/mL MSU crystals for additional 24 h. The control group was treated with vehicle control. The mitochondrial inner transmembrane potential and mitochondrial mass were measured by staining with MitoTracker Deep Red and MitoTracker Green (**A**). The mitochondrial ROS generation was measured by staining with MitoSOX (**B**). The mitochondrial mass was measured by staining with JC-1 and detecting by confocal microscope (**C**) and flow cytometry (**D**).

Mitochondrial ROS and DNA released from damaged mitochondria activate the NLRP3 inflammasome [19]. These findings prompted us to investigate whether 4-HAB inhibits the NLRP3 inflammasome by reducing mitochondrial damage. Using mitochondria-specific labels that distinguished intact (MitoTracker Deep Red) and total (MitoTracker Green) mitochondria, we found that MSU crystals treatment resulted in a reduction in MitoTracker Deep Red staining, indicating obvious mitochondrial damage, but this effect was abolished by 4-HAB (Figure 2A). Additionally, the production of mitochondrial ROS was assessed using MitoSOX, a fluorescent indicator specific for ROS-generating mitochondria. MSU crystals treatment increased mitochondrial ROS production; however, this effect was further increased by 4-HAB (Figure 2B). Furthermore, the results of JC-1 staining (JC-1 FL2) showed that the mitochondrial mass was increased by 4-HAB, as measured by confocal microscopy (Figure 2C) and flow cytometry (Figure 2D).

### 3.3. 4-HAB Reduced Lysosomal Rupture and ASC Oligomerization

**Figure 3:**
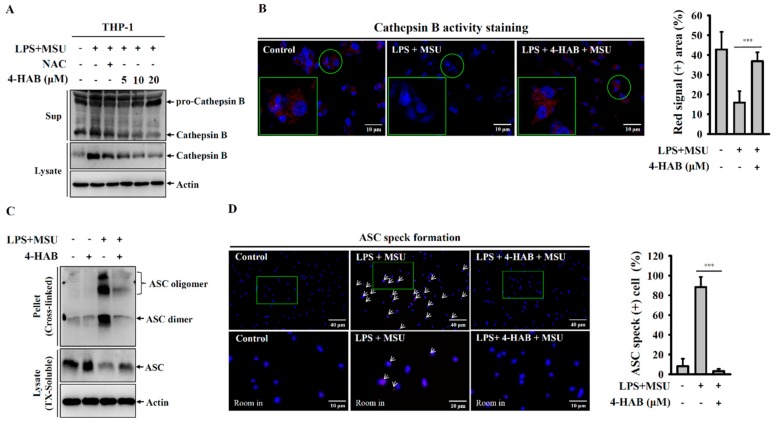
4-HAB reduced lysosomal rupture and ASC oligermerization. THP-1 macrophages were incubated with 1 µg/mL LPS for 5 h followed by incubated with 4-HAB or 10 mM NAC for 30 min. Cells then incubated with 100 μg/mL MSU crystals for additional 24 h. The control group was treated with vehicle control. (**A**) The expression levels of Cathepsin B in the supernatants and cell lysates were measured by Western blot. (**B**) The enzymatic activity of Cathepsin B in the cells was measured by Magic Red Cathepsin B detection kit. (**C**) The levels of ASC oligomerization was measured by Western blot after lysates cross-linked with disuccinimidylsuberate. (**D**) The ASC speck formation was assayed by fluorescent microscope. *** indicates a significant difference at the level of *p* < 0.001 as indicated. (two-tailed *t* test). “+” indicates with; “−“ indicates without.

A previous study showed that lysosomal rupture increases the maturation and release of protease cathepsin B, which in turn activates the NLRP3 inflammasome [20]. We found that MSU crystals increased the release of mature cathepsin B and that these effects were reduced by 4-HAB, indicating reduced lysosomal rupture (Figure 3A). To further verify the protective effect of 4-HAB on lysosomes, the cathepsin B substrate was used to monitor cathepsin B activity. In untreated THP-1 macrophages, the fluorescent signal was bright, which indicated high cathepsin B activity (Figure 3B). However, the fluorescent signal was markedly decreased in MSU crystals-treated cells, which indicated the leakage of cathepsin B into the cytoplasm, and this effect was attenuated by 4-HAB (Figure 3B). Furthermore, activation of the NLRP3 inflammasome results in the recruitment of ASC proteins and the formation of high-molecular-weight oligomers, which in turn activates caspase-1 [21]. To investigate whether 4-HAB is involved in the regulation of ASC oligomerization, the disuccinimidyl suberate-cross-linked cytosolic fractions of THP-1 macrophage lysates were examined by Western blot. In agreement with previous reports, stimulation with MSU crystals was shown to induce the formation of ASC dimers and oligomers. However, 4-HAB significantly abolished ASC dimer and oligomer formation (Figure 3C). Alternatively, ASC oligomerization was also observed by the formation of ASC specks via microscopy. ASC specks induced by MSU crystals were diminished in the presence of 4-HAB (Figure 3D).

### 3.4. 4-HAB Attenuated the NLRP3 Inflammasome through Autophagy Induction

**Figure 4:**
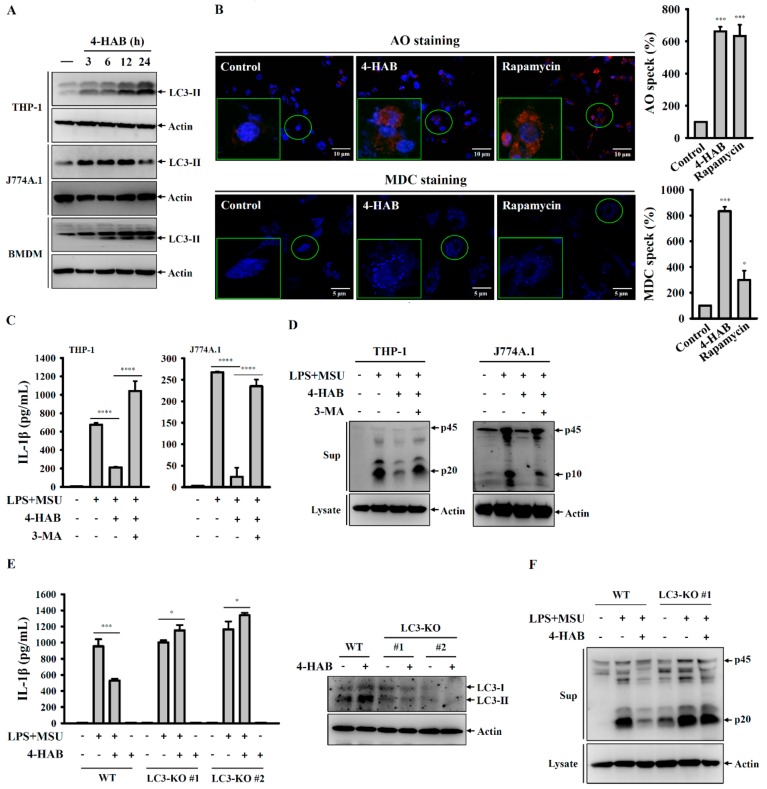
4-HAB attenuated the NLRP3 inflammasome through autophagy induction. (**A**) Cells were incubated with 20 µM 4-HAB for 3–24 h. The expression levels of LC3 in the cell lysates were measured by Western blot. (**B**) THP-1 macrophages were incubated with 20 µM 4-HAB for 24 h or 100 nM rapamycin for 4 h. The fluorescent signals of acridin orange (AO) and MDC were measured by confocal microscope. (**C**,**D**) Cells were incubated with 1 µg/mL LPS for 5 h followed by incubated with 5 mM 3-MA or 20 µM 4-HAB for 30 min. Cells then were incubated with 100 μg/mL MSU crystals for additional 24 h. The levels of IL-1β (**C**) and caspase-1 (**D**) in the supernatants were measured by ELISA and Western blot, respectively. (**E**,**F**) Wild-type and LC3-knockout THP-1 macrophages were incubated with 20 µM 4-HAB or vehicle for 24 h. The levels of LC3 in the cell lysates were measured by Western blot (**E**, right panel). Wild-type and LC3-knockout THP-1 macrophages were incubated with 1 µg/mL LPS for 5 h followed by incubated for 30 min with 20 µM 4-HAB. Cells then were incubated with 100 μg/mL MSU crystals for additional 24 h. The levels of IL-1β (**E**, left panel) and caspase-1 (**F**) in the supernatants were measured by ELISA and Western blot, respectively. The control group was treated with vehicle control. The ELISA data are expressed as the mean ± SD of three separate experiments. *, *** and **** indicate a significant difference at the level of *p* < 0.05, *p* < 0.001 and *p* < 0.0001, respectively, compared to untreated cells (**B**) or as indicated. (One-way ANOVA with Dunnett’s multiple comparisons test in (**B**) or two-tailed *t* test in (**C**) and (**E**). “+” indicates with; “−“ indicates without.

Autophagy is a self-protective cellular process that plays a housekeeping role by removing damaged proteins and organelles. Accumulating evidence indicates that autophagy negatively regulates the NLRP3 inflammasome [22]. Thus, we examined whether suppression of the NLRP3 inflammasome by 4-HAB is mediated by autophagy induction. Western blot analysis revealed that incubation with 4-HAB time-dependently increased expression levels of the autophagy induction marker LC3-II in THP-1 macrophages, J774A.1 macrophages and BMDM (Figure 4A). Autophagy induction is also characterized by the formation of acidic vesicular organelles that can be examined by AO and MDC staining [22]. By AO and MDC staining, we found that 4-HAB increased the accumulation of acidic vesicular organelles in THP-1 macrophages (Figure 4B). These results were comparable with the results obtained with the known autophagy inducer rapamycin (Figure 4B). To clarify the role of autophagy in suppression of the NLRP3 inflammasome, the known autophagy inhibitor 3-MA was added to THP-1 and J774A.1 macrophages. 3-MA restored IL-1β secretion (Figure 4C) and caspase-1 activation (Figure 4D) in 4-HAB-treated THP-1 and J774A.1 macrophages. To further confirm the effect of autophagy on 4-HAB-mediated NLRP3 inflammasome inhibition, we generated LC3-knockout THP-1 macrophages by the CRISPR/Cas9 system. 4-HAB failed to inhibit MSU crystals-induced IL-1β secretion (Figure 4E) and caspase-1 activation (Figure 4F) in LC3-knockout THP-1 macrophages. These results indicated that 4-HAB inhibits the NLRP3 inflammasome through autophagy induction.

### 3.5. 4-HAB Inhibited the NLRP3 Inflammasome by Enhancing the Sirt1/Autophagy Axis

**Figure 5:**
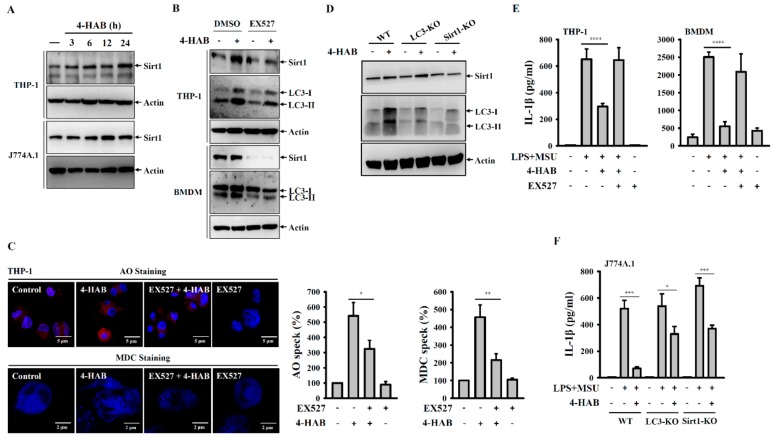
4-HAB inhibited the NLRP3 inflammasome by enhancing the Sirt1/autophagy axis. (**A**) Cells were incubated with 20 µM 4-HAB for 3–24 h. The expression levels of Sirt1 in the cell lysates measured by Western blot. (**B**,**C**) Cells were incubated with 10 µM EX527 or DMSO for 24 h followed by incubated with 20 µM 4-HAB or vehicle for 24 h. The expression levels of Sirt1 and LC3 in the cell lysates measured by Western blot (**B**). The fluorescent signals of AO and MDC were measured by confocal microscope (**C**). (**D**) Wild-type, LC3-knockout and Sirt1-knockout J774A.1 macrophages were incubated with 20 µM 4-HAB or vehicle for 24 h. The expression levels of LC3 and Sirt1 in the cell lysates were measured by Western blot. (**E**) Cells were incubated with 1 µg/mL LPS for 5 h followed by incubated with 10 µM EX527 or 20 µM 4-HAB for 30 min. Cells then were incubated with 100 μg/mL MSU crystals for additional 24 h. The expression levels of IL-1β in the supernatants were measured by ELISA. (**F**) Wild-type, LC3-knockout and Sirt1-knockout J774A.1 macrophages were incubated with 1 µg/mL LPS for 5 h followed by incubated with 20 µM 4-HAB for 30 min. Cells then were incubated with 100 μg/mL MSU crystals for additional 24 h. The expression levels of IL-1β in the supernatants were measured by ELISA. The control group was treated with vehicle control. The ELISA data are expressed as the mean ± SD of three separate experiments. *, **, *** and **** indicate a significant difference at the level of *p* < 0.05, *p* < 0.01, *p* < 0.001 and *p* < 0.0001, respectively, as indicated. (two-tailed *t* test). “+” indicates with; “−“ indicates without.

Autophagy activity has been reported to be upregulated by the high expression of Sirt1 [23,24]. To further investigate the mechanisms of 4-HAB-mediated autophagy induction and NLRP3 inflammasome inhibition, the effect of 4-HAB on Sirt1 expression was investigated. 4-HAB time-dependently enhanced Sirt1 expression in both THP-1 and J774A.1 macrophages (Figure 5A). Next, we found that the Sirt1 inhibitor EX527 reduced 4-HAB-mediated Sirt1 and LC3-II expression in THP-1 macrophages and BMDM (Figure 5B). Additionally, EX527 reduced AO and MDC staining in 4-HAB-treated THP-1 cells (Figure 5C). To confirm the effect of Sirt1 on 4-HAB-mediated autophagy induction, we generated Sirt1- and LC3-knockout J774A.1 macrophages by the CRISPR/Cas9 system. 4-HAB failed to induce LC3-II expression in Sirt1-knockout J774A.1 cells (Figure 5D). These results indicated that Sirt1 is found upstream of autophagy in 4-HAB-treated cells. Notably, inhibition of Sirt1 by EX527 restored IL-1β secretion in 4-HAB-treated THP-1 macrophages and BMDM (Figure 5E). In addition, the IL-1β inhibitory activity of 4-HAB was reduced in Sirt1- and LC3-knockout J774A.1 macrophages (Figure 5F). Taken together, these results indicated that 4-HAB inhibits MSU crystals-induced activation of the NLRP3 inflammasome through the Sirt1/autophagy axis.

### 3.6. 4-HAB Inhibited NLRP3 Inflammasome Activation in a Mouse Model of Gouty Inflammation

**Figure 6:**
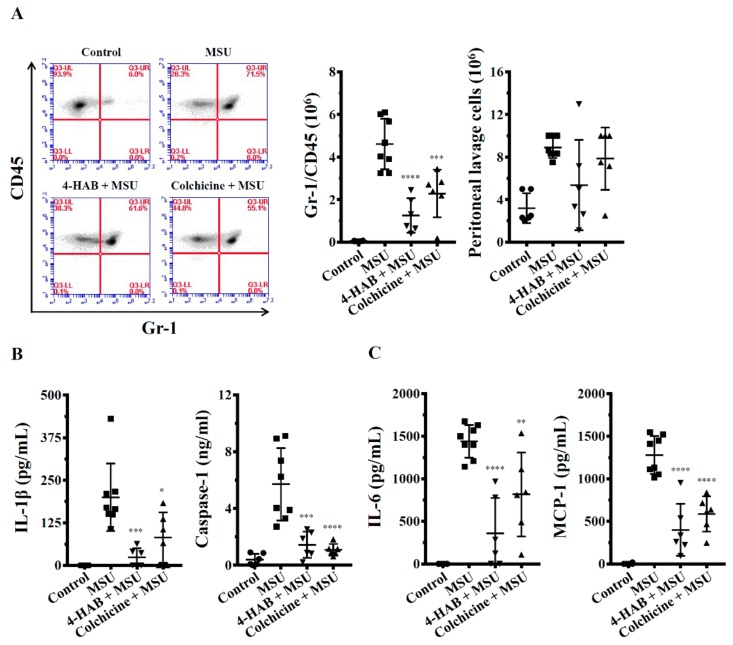
4-HAB inhibited NLRP3 inflammasome activation and reduced inflammation in a mouse model of MSU crystals-mediated peritonitis. (**A**) Neutrophil and peritoneal lavage cells influx quantified by Gr-1 and CD45 staining and cell count, respectively. (**B**,**C**) The expression levels of IL-1β, active caspase-1, IL-6 and MCP-1 in the peritoneal lavage fluids were measured by ELISA. The ELISA data are expressed as mean ± SD of three separate experiments. *, **, *** and **** indicate a significant difference at the level of *p* < 0.05, *p* < 0.01, *p* < 0.001 and *p* < 0.0001, respectively, compared to MSU crystals-injected mice. (One-way ANOVA with Dunnett’s multiple comparisons test).

Finally, we evaluated whether 4-HAB attenuates disease development by inhibiting the NLRP3 inflammasome in vivo. NLRP3 inflammasome-associated mouse peritonitis is triggered by the intraperitoneal injection of MSU crystals [25]. We found that neutrophil recruitment and NLRP3 inflammasome activation in the peritoneum were significantly increased in mice treated with MSU crystals compared to those in the control mice. However, the treatment of mice by the i.p. administration of 4-HAB (20 mg/kg body weight) or colchicine (1 mg/kg body weight) significantly blocked neutrophil flux (Figure 6A) and reduced the levels of IL-1β and active caspase-1 in the peritoneal lavage fluid (Figure 6B). Furthermore, mice treated with 4-HAB or colchicine had lower IL-6 and MCP-1 levels in peritoneal lavage fluid compared to those in MSU crystals-treated mice (Figure 6C). These results indicated that 4-HAB suppresses NLRP3 inflammasome activation and inflammation in vivo.

## 4. Discussion

Gouty inflammation is characterized by intense pain caused by the deposition of MSU crystals into the articular joint and surrounding tissues [25]. Uptake of MSU crystals by macrophages induces proinflammatory cytokine and chemokine productions, which leads to the further recruitment of immune cells into the joint and escalates the inflammation [25,26]. The current therapeutic approaches against gouty inflammation are given mainly to reduce hyperuricaemia and inflammatory status [25]. For instance, allopurinol reduces the serum uric acid levels by inhibiting xanthine oxidase, but it is unable to reduce the inflammation during acute phase and may cause fever, skin rashes, allergic reactions, hepatitis and nephropathy [5,25]. Thus, non-steroidal anti-inflammatory (NSAID) drugs (e.g., indomethacin) and alkaloid drugs (e.g., colchicine) are frequently used as first-line therapies for the treatment of acute gouty inflammation [25]. In addition, it has been demonstrated that the NLRP3 inflammasome plays important roles in MSU crystals-induced gouty inflammation [27,28]. Targeting NLRP3 inflammasome produced IL-1β and IL-18 by recombinant IL-1 receptor antagonist (anakinra), neutralizing IL-1β antibody (canakinumab), soluble decoy IL-1 receptor (rilonacept), IL-18-binding protein and anti-IL-18 receptor antibody are also useful in the treatment of gouty inflammation [5]. However, the high cost of such treatments limits their wide clinical use. Novel therapeutic agents for gouty inflammation are therefore needed. As part of our study programme to identify novel therapeutic agents for the NLRP3-asscoiated complications, herein we investigated the effects of 4-HAB on NLRP3 inflammasome and the finding of this study clearly show that 4-HAB inhibited MSU crystals-induced NLRP3 inflammasome activation in intro and in vivo.

NLRP3 inflammasome activation results in not only IL-1β and IL-18 secretion but also pyroptosis, a caspase-1-dependent cell death characterized by the loss of membrane integrity [18]. Notably, the inflammasome component ASC is released from the membrane damage macrophages upon NLRP3 inflammasome activation and amplify the inflammatory response by stimulating the surrounding cells [29]. We found that 4-HAB reduced the PI uptake in MSU crystals-activated macrophages, indicating the reduced membrane integrity loss (Figure 1F). In addition, 4-HAB also inhibited MSU crystals-induced ASC release (Figure 1C), suggesting that 4-HAB can prevent the inflammatory response amplified by the release ASC [29]. These data suggest that 4-HAB inhibits pyroptosis in MSU crystals-activated macrophages.

Mitochondrial damage induced by activation signal is one of the vital events for the NLPR3 inflammasome activation [19,20]. The uptake of MSU crystals by macrophages induces mitochondrial ROS generation and increases the oxidative status of mitochondrial DNA. The oxidized mitochondrial DNA release into cytosol because of the mitochondrial membrane integrity loss. The oxidized mitochondrial DNA binds to NLRP3, promotes NLRP3 inflammasome assembly and activates the NLRP3 inflammasome [25]. Although a low level of ROS promotes NLRP3 inflammasome activation, a recent study demonstrated that a high level of ROS generated by Streptococcus pneumonia infection inhibited the NLRP3 inflammasome through the oxidation of the inflammasome components ASC and caspases [30]. We found that although 4-HAB increased mitochondrial ROS production (Figure 2B), it significantly reduced mitochondrial integrity loss induced by MSU crystals (Figure 2A). These results indicated that 4-HAB inhibits the NLRP3 inflammasome through reduced oxidized mitochondrial DNA release and that a high level of mitochondrial ROS induced by 4-HAB may induce oxidation of NLRP3 inflammasome components, which contributes to 4-HAB-mediated NLRP3 inflammasome inhibition. Phagocytosis of MSU crystals by macrophages results in lysosomal rupture, and leads to the release of cathepsin B, a known activator of the NLRP3 inflammasome [27,28]. We found that 4-HAB protected against lysosomal rupture and reduced cathepsin B release (Figure 3A,B). Lysosomal rupture also activates the TAK1-JNK pathway, which is necessary for complete activation of the NLRP3 inflammasome through the oligomerization of ASC [31]. We demonstrated that 4-HAB reduced the oligomerization of ASC (Figure 3C,D); however, the effect of 4-HAB on the activation of TAK1-JNK needs further investigation. Notably, our previous study showed that 4-HAB inhibited proIL-1β in LPS-activated macrophages; however, 4-HAB did not inhibit NLRP3 expression [10]. These results indicated that 4-HAB inhibited MSU-mediated NLRP3 inflammasome activation was mainly through reducing the activation signal, but not through inhibiting the LPS-mediated priming signal.

Autophagy is a self-protective mechanism of the cells that maintains the cellular homeostasis by removing or recycling intracellular dysfunctional components [32]. The enzyme converts pro-LC3B to LC3B-I by cleaving its C-terminus, after which LC3B-I can be conjugated to phosphatidylethanolamine by cysteine protease, thereby forming LC3B-II. This characteristic conversion of endogenous LC3-I to LC3-II is an autophagy biomarker that is used to monitor autophagy activity [32]. Autophagy negatively regulates the NLRP3 inflammasome through removing damaged mitochondria to prevent the release of mitochondrial ROS and mitochondrial DNA into the cytoplasm, ultimately limiting NLRP3 inflammasome assembly [32,33]. NLRP3 inflammasome inhibition and autophagy induction is cooperative and of great relevance for the development of novel therapeutic strategies against NLRP3-asscoiated complications. We demonstrated that 4-HAB is a novel autophagy inducer that attenuates gouty inflammation by inhibiting the NLRP3 inflammasome. In addition, Sirt1, a class III histone deacetylase, is generally known as a vital regulator of inflammatory processes [34]. Sirt1 inhibits the NLRP3 inflammasome activation in mesenchymal stem cells and vascular endothelial cells [35,36]. Sirt1 activator treatment significantly suppressed caspase-1 activation in mouse macrophages, linking Sirt1 to regulation of the NLRP3 inflammasome [37]. Furthermore, Sirt1 inhibited TNF-α-induced IL-1β production through the autophagy-dependent degradation of NLRP3 in vascular adventitial fibroblasts [23]. Consistent with these observations, our results showed that 4-HAB increased Sirt1 expression (Figure 5A), and the Sirt1 inhibitor EX527 and Sirt1 knockout diminished autophagy induction and the inhibitory effects of 4-HAB on the NLRP3 inflammasome (Figure 5E,F). Although we have gained promising data concerning the protective effect of Sirt1 on 4-HAB-mediated NLRP3 inflammasome inhibition, further studies are needed to address the molecular mechanism underlying 4-HAB-mediated regulation of Sirt1 expression.

**Figure 7:**
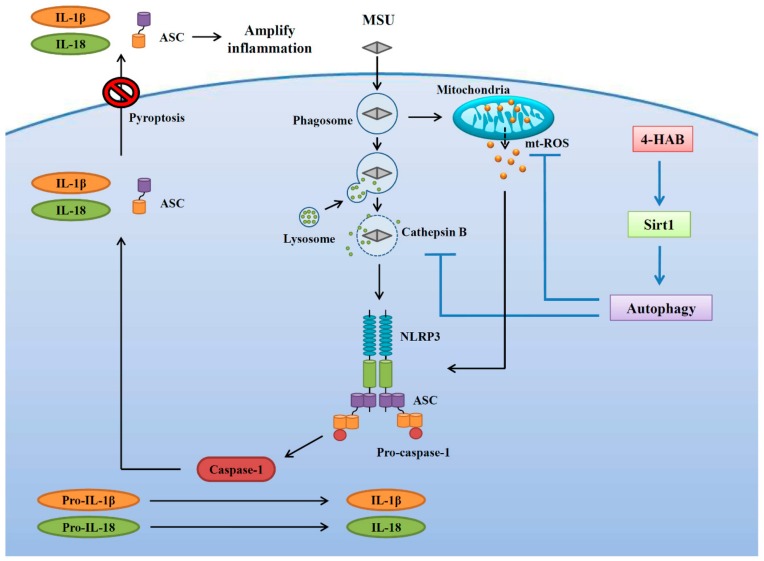
Overview of the demonstrated and putative mechanisms by which 4-HAB attenuated the NLRP3 inflammasome.

In conclusion, this study identified 4-HAB as an anti-NLRP3 inflammasome agent. Specifically, 4-HAB attenuated MSU crystals-mediated NLRP3 inflammasome activation by blocking mitochondrial damage, lysosomal rupture-mediated cathepsin B release and ASC oligomerization. Furthermore, 4-HAB inhibited the NLRP3 inflammasome by increasing the Sirt1/autophagy axis. The inhibitory effects of 4-HAB on the NLRP3 inflammasome were further confirmed in a mouse model of MSU crystals-induced peritonitis. A schematic representation of the present study is shown in Figure 7. Thus, 4-HAB is a potential pharmacological agent for the management of gouty inflammation. However, the limitation of this study is the lack of toxicity test in a mice model. Although we did not observe any toxic response in our current mice study, a long term 4-HAB treatment or a high dosage 4-HAB treatment in mice should be conducted to verify the in vivo safety. Another imitation of this study is only using the mouse model of MSU crystals-induced peritonitis to test the in vivo activity of 4-HAB, it is better to confirm the anti-gouty inflammation activity of 4-HAB using a subcutaneous air pouch model in mouse.

## 5. Conclusions

4-HAB is a novel autophagy inducer that attenuates gouty inflammation by inhibiting the NLRP3 inflammasome in vitro and in vivo.
